# Adeno-associated Virus-mediated Ezh2 Knockdown Reduced the Increment of Newborn Neurons Induced by Forebrain Ischemia in Gerbil Dentate Gyrus

**DOI:** 10.1007/s12035-024-04200-w

**Published:** 2024-04-27

**Authors:** Yoshihide Sehara, Yuki Hashimotodani, Ryota Watano, Kenji Ohba, Ryosuke Uchibori, Kuniko Shimazaki, Kensuke Kawai, Hiroaki Mizukami

**Affiliations:** 1 Division of Genetic Therapeutics, Center for Molecular Medicine, Jichi Medical University, 3311-1 Yakushiji, Shimotsuke, Tochigi, 329-0498 Japan; 2 Graduate School of Brain Science, Doshisha University, Kyotanabe, Japan; 3 Department of Neurosurgery, Jichi Medical University, Shimotsuke, Japan

**Keywords:** Adeno-associated virus vector, Gerbil, Neurogenesis, Polycomb group complex, Transient forebrain ischemia

## Abstract

It is established that neurogenesis of dentate gyrus is increased after ischemic insult, although the regulatory mechanisms have not yet been elucidated. In this study, we focused on Ezh2 which suppresses gene expression through catalyzing trimethylation of lysine 27 of histone 3. Male gerbils were injected with adeno-associated virus (AAV) carrying shRNA targeting to Ezh2 into right dentate gyrus 2 weeks prior to forebrain ischemia. One week after ischemia, animals were injected with thymidine analogue to label proliferating cells. Three weeks after ischemia, animals were killed for histological analysis. AAV-mediated knockdown of Ezh2 significantly decreased the ischemia-induced increment of proliferating cells, and the proliferated cells after ischemia showed significantly longer migration from subgranular zone (SGZ), compared to the control group. Furthermore, the number of neural stem cells in SGZ significantly decreased after ischemia with Ezh2 knockdown group. Of note, Ezh2 knockdown did not affect the number of proliferating cells or the migration from SGZ in the non-ischemic condition. Our data showed that, specifically after ischemia, Ezh2 knockdown shifted the balance between self-renewal and differentiation toward differentiation in adult dentate gyrus.

**Supplementary Information:**

The online version contains supplementary material available at 10.1007/s12035-024-04200-w.

## Introduction

Out-of-hospital cardiac arrest is a serious problem which causes severe neurological deficit and death. Survival of out-of-hospital cardiac arrest ranges from 8 to 16% [1–3], among which neurocognitive sequelae are reported to be prevalent with a range from 6 to 100% [4, 5]. A common pattern of neurocognitive sequelae is memory impairment [6]. Although immediate resuscitation and therapeutic hypothermia have been improving the prognosis of cardiac arrest, the high prevalence of sequelae limits the social activity after discharge.

Neurons had not been believed to be newly generated in adult human brain until Eriksson et al. demonstrated that neurogenesis takes place in the two distinct regions of adult human brains including subventricular zone (SVZ) of lateral ventricle and dentate gyrus (DG) of hippocampus in 1998 [7]. Furthermore, the neurogenesis in SVZ and DG is proved to be increased after ischemic insult [8–10]. In addition, it has been established that the neurogenesis is also increased by other neurodegenerative status, such as epilepsy and trauma [11–13]. Although it should be an intriguing phenomenon that neurogenesis is increased in these neurodegenerative status, the mechanisms and roles of neurogenesis remain unelucidated.

Hippocampus plays a pivotal role of cognitive functions such as learning and memory. In DG of hippocampus, neural stem cells of SGZ give rise to mature granule cells, which are the principal neurons in the DG. In the hippocampal circuit, granule cells receive input from entorhinal cortex and send their output to CA3 region along the mossy fiber tract [14]. While granule cells play an important role in the hippocampal information processing, little is known about the relationship between neurocognitive sequelae of cardiac arrest and the post-ischemic increase of neurogenesis in hippocampus, which can be a cue to solve the problem.

In this study, we focused on the regulatory system of post-ischemic increase of neurogenesis in DG. Especially, the main focus of this study is the role of Ezh2 which suppresses gene expression through catalyzing trimethylation of lysine 27 of histone 3 (H3K27me3) [15]. Ezh2 is a catalytic unit of polycomb repressive complex 2 (PRC2) which forms a large protein complex with EeD and Suz12, while Ringlb and Bmi1 form core of PRC1 [16]. The balance between self-renewal and differentiation of neural stem cells is partly regulated by epigenetic repression. Previous studies reported that Ezh2 contributes to the regulation of this balance. Ezh2 knockdown was reported to disrupt the neural tube integrity and reduce neural stem cells in the chick embryo [17]. Also, cortex-specific knockout of Ezh2 shifted the balance between self-renewal and differentiation toward differentiation in the mice embryonic corticogenesis [18]. Cerebellar deletion of Ezh2 increased GABAergic interneurons and decreased Purkinje cells in conditional knockout mice embryo [19]. In contrast to these studies investigating the roles of Ezh2 in embryo, there are few studies addressing the role of Ezh2 on adult neurogenesis. Concerning adult mice, conditional knockout of Ezh2 in the neural stem cells of SVZ showed marked reduction of adult neurogenesis, suggesting that Ezh2 is necessary for the maintenance of stem cell neuronal lineages [20]. However, there is no report which focuses on roles of Ezh2 in adult neurogenesis in neurodegenerative diseases. Here, we show the regulatory roles of Ezh2 on adult neurogenesis of DG after transient forebrain ischemia using adult gerbils.

## Material and Methods

### shRNA Cloning and Screening

**Fig. 1:**
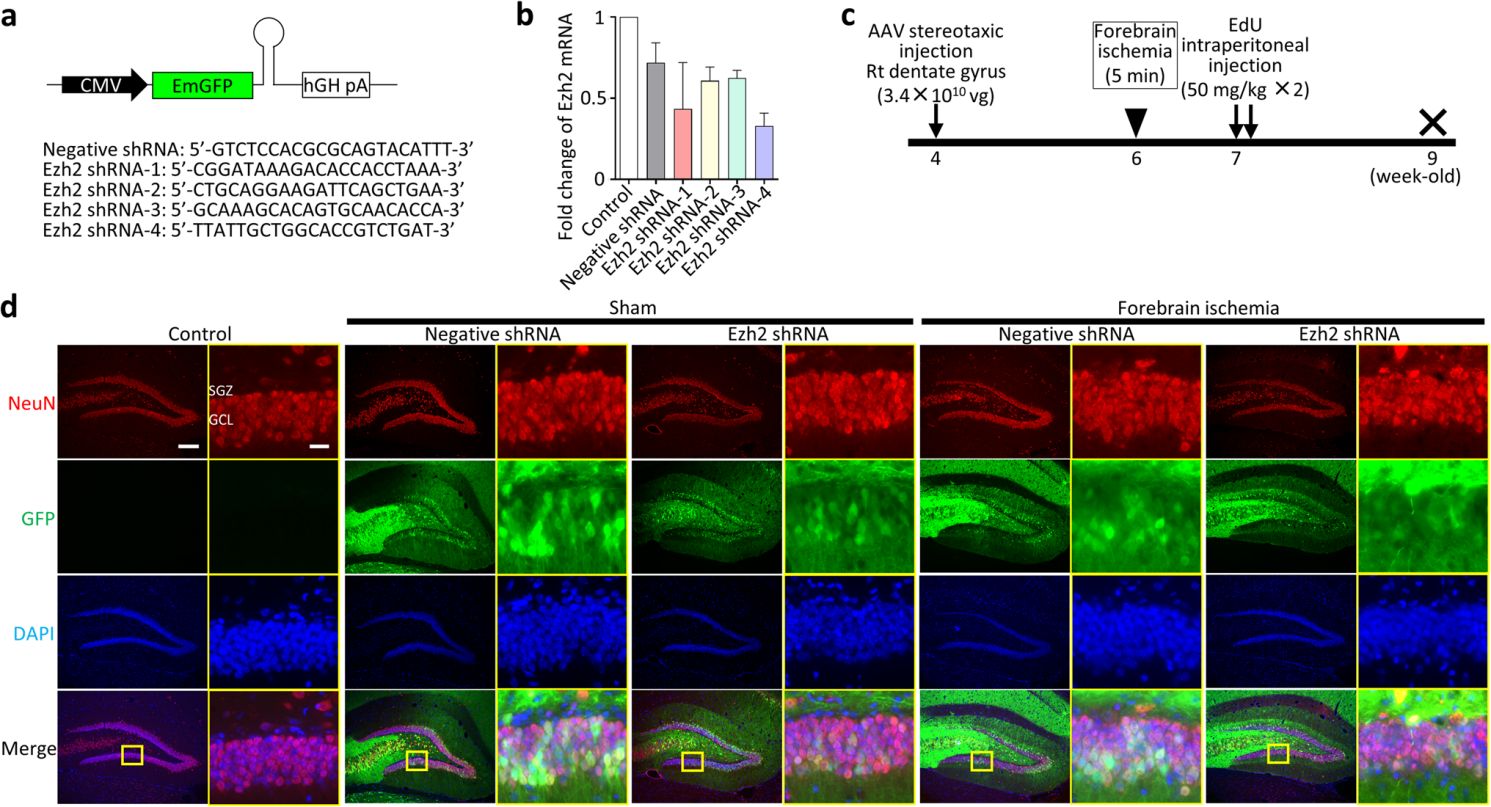
AAV vector carrying Ezh2 shRNA was expressed whole DG of hippocampus. **a** Construct of RNA-mediated gene silencing system delivered by AAV vector. **b** Four different shRNA constructs were transduced in gerbil fibroma cells for 48 h. Ezh2 shRNA-4 decreased Ezh2 expression by 60–70%. **c** Experimental design of this study. **d** Representative images of DG treated with Ezh2 shRNA-4 3 weeks after transient forebrain ischemia. The mature neuron marker, NeuN, shows the granule cell layer. The GFP signals were prominent in cell bodies and dendrites of neurons. AAV, adeno-associated virus; CMV, cytomegalovirus promoter; EdU, 5-ethynyl-2′-deoxyuridine; EmGFP, emerald green fluorescent protein; DAPI, 4′,6-diamidino-2-phenylindole; GCL, granule cell layer; SGZ, subgranular zone. Scale bar, 200 μm in low magnification images and 20 μm in high magnification images (in the yellow rectangulars). Data represent *mean* ± *SD*

shRNA sequences targeting gerbil *Ezh2* were designed using an online tool (BLOCK-iT RNAi Designer by Thermofisher Scientific, https://rnaidesigner.thermofisher.com/rnaiexpress/) and were subcloned into pcDNA™6.2-GW/EmGFP-miR plasmid (Thermofisher Scientific, Waltham, MA; Fig. 1a): *Ezh2* shRNA-1 (5′-CGGATAAAGACACCACCTAAA-3′); *Ezh2* shRNA-2 (5′-CTGCAGGAAGATTCAGCTGAA-3′); *Ezh2* shRNA-3 (5′-GCAAAGCACAGTGCAACACCA-3′); *Ezh2* shRNA-4 (5′-TTATTGCTGGCACCGTCTGAT-3′); and negative shRNA, which is predicted not to target any vertebrae gene (5′-GTCTCCACGCGCAGTACATTT-3′). The efficacy of *Ezh2* shRNA was evaluated by real-time PCR 48 h after transfection into IMR-33 gerbil fibroma cells (CCL-146™, American Type Culture Collection) using Lipofectamine 3000 (Thermofisher Scientific; Fig. 1b). RNA was extracted using RNeasy Mini kit (Qiagen, Hilden, Germany). After RNA concentration was measured by means of a Nanodrop 1000 (Thermofisher Scientific), aliqots of RNA (1 μg) were reverse-transcribed to cDNA using PrimeScript™ RT reagent Kit with gDNA Eraser (Takara Bio, Kusatsu, Japan). Real-time PCR amplification of *Ezh2* was performed in triplicate using gene-specific primers and TB Green® Premix Ex Taq™ II (Takara Bio): *Ezh2* forward (5′-AGGCTGGGGGATTTTTATCA-3′) and *Ezh2* reverse (5′-TGCATCCACCACAAAATCAT-3′). As an internal control for normalization, PCRs were performed with the amplification of the reference gene *Gapdh*: *Gapdh* forward (5′-CAACAGTGGCAAAGTGGAGA-3′) and *Gapdh* reverse (5′-GATCTCGCTCCTGGAAGATG-3′).

### AAV Production

For RNAi-mediated silencing of Ezh2 in intact animals, we cloned shRNA in the pcDNA™6.2-GW/EmGFP-miR plasmid into the pAAV-hrGFP plasmid with double digestion using SacI and XhoI (Agilent Technologies, Santa Clara, CA). Recombinant AAV-CMV-Ezh2 shRNA and AAV-CMV-negative shRNA were prepared as previously described [21]. Briefly, 60% confluent human embryonic kidney cells (HEK293; Agilent Technologies) were incubated in large culture vessels and cotransfected with an AAV vector plasmid, adenoviral helper plasmid pHelper (Agilent Technologies), and pAAV2 Rep/AAVrh10. Crude viral lysates were purified using an AAV purification kit (Takara Bio). Viral titers were determined by real-time PCR in triplicate using gene-specific CMV promoter primers and TB Green® Premix Ex Taq™ II (Takara Bio): CMV promoter forward (5′-CATTATGCCCAGTACATGACC-3′) and CMV promoter reverse (5′-GTGAGTCAAACCGCTATCC-3′). The viral stock in this study was diluted with phosphate buffer saline (PBS) and adjusted to the final concentration of 1.7 × 10^10^ viral genomes (vg)/μl.

### Stereotaxic Injection

Animal experiments were carried out in a humane manner after receiving approval from the Institutional Animal Experiment Committee of the Jichi Medical University. Gerbils were maintained in ventilated cages with 12 h day/night cycles and access to food and water ad libitum. Twenty-three male gerbils (4 weeks old, body weight: 28–40 g, from Japan SLC, Hamamatsu, Japan) were anesthetized with 2% isoflurane in a mixture of nitrous oxide and oxygen (70:30) and placed in a stereotaxic apparatus (Narishige Scientific Instrument Lab., Tokyo, Japan). AAV-CMV-Ezh2 shRNA (*n* = 12) or AAV-CMV-negative shRNA (*n* = 11) viral stocks were injected at a rate of 0.2 μl/min through a 26-gauge Hamilton microsyringe (Hamilton, Reno, NV) mounted on a micromanipulator (Narishige). Stereotaxic injection coordinates for DG of hippocampus were 2.0 mm caudal to the bregma, 1.5 mm right to the midline, and 2.5 mm below the pial surface with a 2 μl injection (3.4 × 10^10^ vg in total). The needle remained in place for 5 additional minutes before the cannula was slowly withdrawn to prevent reflux. The skin incision was closed with a suture, and the rectal temperature was monitored and maintained at 37 ± 0.5 °C using a heat blanket (Bio Research Center, Nagoya, Japan) until being returned to the cage. For the control group, animals did not receive AAV or vehicle injection (*n* = 5).

### Global Ischemia

Two weeks after AAV injection, gerbils (6 weeks old, body weight 48–54 g) were subjected to transient forebrain ischemia by two-vessel occlusion (5 min) or sham operation, as described previously [22]. Briefly, a ventral midline neck incision was made under anesthesia with 2% isoflurane in a mixture of nitrous oxide and oxygen (70:30). Both carotid arteries were occluded with aneurysm clips (Mizuho, Tokyo, Japan) for 5 min. The rectal temperature was monitored and maintained at 37.5 ± 0.5 °C using a heat blanket (Bio Research Center) until being returned to the cage. Three animals died during surgical procedure (*n* = 10 after surgeries; *n* = 5 for AAV-CMV-Ezh2 shRNA group; and *n* = 5 for AAV-CMV-negative shRNA). Sham-operated animals were treated identically except that the carotid arteries were not occluded after the neck incision (*n* = 10 in total; *n* = 5 for AAV-CMV-Ezh2 shRNA group, and *n* = 5 for AAV-CMV-negative shRNA).

### 5-Ethynyl-2′-Deoxyuridine (EdU) Labeling

**Fig. 2:**
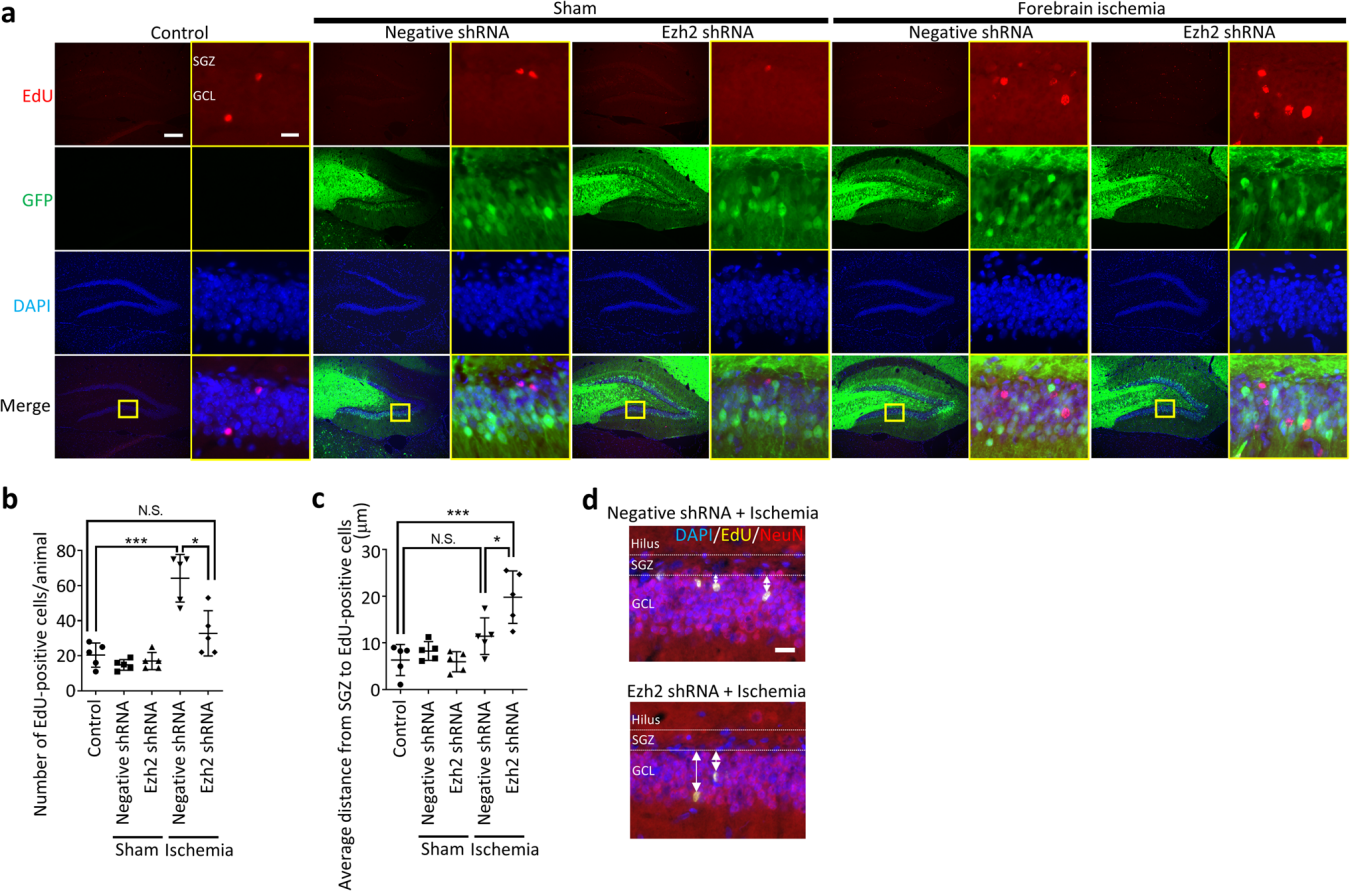
Ezh2 knockdown abolished increment of neurogenesis and promoted neural differentiation after transient forebrain ischemia. **a** Representative images of dentate gyrus treated with Ezh2 shRNA-4 3 weeks after transient forebrain ischemia. EdU-positive cells were prominent in subgranular zone and granule cell layer. **b** Quantification of EdU-positive cells in the dentate gyrus. Transient forebrain ischemia significantly increased EdU-positive cells of the negative shRNA group. On the other hand, Ezh2 knockdown abolished the post-ischemic increase of the EdU-positive cells. **c** Quantification of distances between subgranular zone and EdU-positive cells. Transient forebrain ischemia increased the distance between subgranular zone and EdU-positive cells both in negative shRNA (not significant) and Ezh2 RNA groups (*p* < 0.001). **d** Representative images of measurement method of distances between subgranular zone and EdU-positive cells (two-way arrows). DAPI, 4′,6-diamidino-2-phenylindole; EdU, 5-ethynyl-2′-deoxyuridine; GCL, granule cell layer; GFP, green fluorescent protein; SGZ, subgranular zone. Scale bar = 200 μm in low magnification images (**a**), 20 μm in high magnification images (in the yellow rectangulars, **a**), and 20 μm in **d**. ANOVA followed by Tukey. Data represent *mean* ± *SD*. * *p* < 0.05. *** *p* < 0.001. N.S., not significant

To label newly born cells, gerbils (7 weeks old, weight 54–60 g) received intraperitoneal injection of EdU (50 mg/kg body weight; Tokyo Chemical Industry, Tokyo, Japan) dissolved in PBS 1 week after forebrain ischemia twice every other day (Fig. 1c). Two weeks after EdU injection, animals were deeply anesthetized, and brains were perfused through the left ventricle with 10% neutral buffered formalin. After perfusion-fixation, brains were taken and then immersed in 10% neutral buffered formalin overnight, followed by sucrose cryoprotection for 48 h. After frozen in powdered dry ice, coronal brain sections at 10-μm thickness were made on a sliding microtome. EdU was visualized using Click-iT® plus EdU Alexa Fluor™ 594 (Fig. 2a) or 647 (Fig. 2d) Imaging kit (Thermofisher Scientific). After EdU labeling, the antibody labeling was performed according to the protocol shown in the next section.

### Immunohistochemistry

**Fig. 3:**
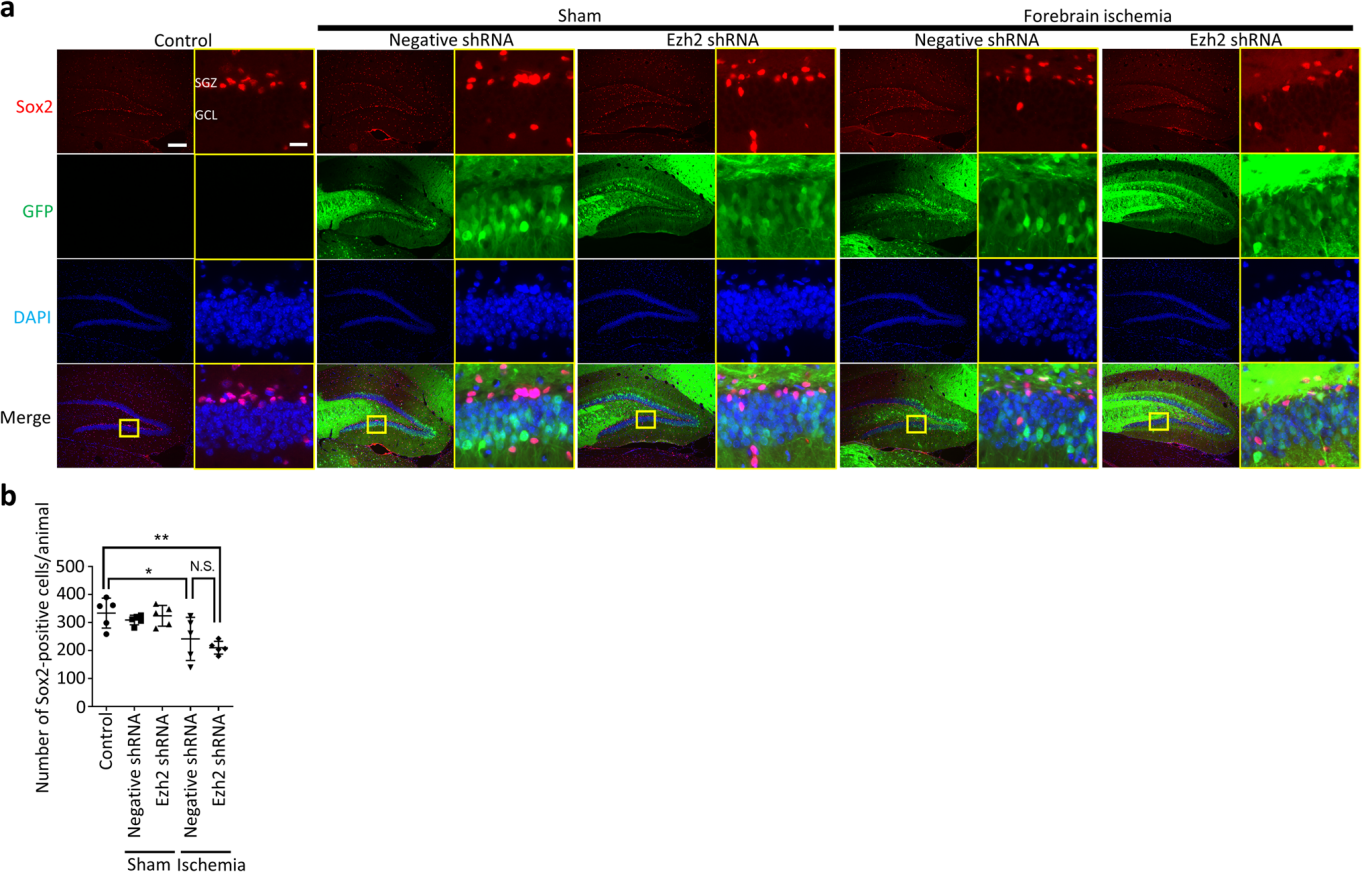
Ezh2 knockdown decreased neural stem cells in SGZ after forebrain ischemia. **a** Representative images of dentate gyrus treated with Ezh2 shRNA-4 3 weeks after transient forebrain ischemia. Sox2-positive cells were prominent in subgranular zone. **b** Quantification of Sox2-positive cells in the dentate gyrus. Transient forebrain ischemia significantly decreased Sox2-positive cells of negative shRNA group (*p* < 0.05) and Ezh2 shRNA group (*p* < 0.01). GCL, granule cell layer; GFP, green fluorescent protein; SGZ, subgranular zone. Scale bar = 200 μm in low magnification images and 20 μm in high magnification images (in the yellow rectangular). ANOVA followed by Tukey. Data represent *mean* ± *SD*. * *p* < 0.05. ** *p* < 0.01. N.S., not significant

Coronal sections (10-μm thickness) were incubated with a primary antibody against NeuN (diluted at 1:1000; MAB377, Merck, Darmstadt, Germany), Sox2 (diluted at 1:1000, ab79351; Abcam, Cambridge, UK) or Ezh2 (diluted at 1:1000; #5246, Cell Signaling, Danvers, MA, USA) in PBS containing 0.3% Triton X-100 at 4 °C overnight. Only for Ezh2, the sections were placed in a jar filled with 0.01 M citrate buffer (pH 6.0) and irradiated in a microwave oven for 10 min before incubation with a primary antibody. After washing with PBS, the sections were incubated with a secondary antibody against anti-mouse IgG (diluted at 1:1000; #4409, Cell Signaling) or anti-rabbit (diluted at 1:1000; A10040, Thermofisher Scientific) for 1 h at room temperature. After washing with PBS, the sections were coverslipped with a mounting agent including 4′6-diamino-2-phenylindole (DAPI; Thermofisher Scientific). Immunoreactivity is assessed using a BZ-9000 microscope (Keyence, Osaka, Japan) in Figs. 1d, 2a, 3a, and Supplemental Figure and a BZ-X810 microscope (Keyence) in Fig. 2d. The EdU- or Sox2-positive cells in the SGZ and granule cell layer (GCL) were automatically counted using the BZ-II analyzer software (Keyence). Twenty micro-meters from the inner surface of the GCL was defined as SGZ. The number of immuno-positive cells of one animal was defined as the mean of three sections at 1.7, 2.0, and 2.3 mm caudal to the bregma. In Fig. 2c, the average distance from SGZ to EdU-positive cells of one animal is defined as the mean of the distances vertical to the SGZ of all EdU-positive signals of three sections at 1.7, 2.0, and 2.3 mm caudal to the bregma (Fig. 2d).

### Statistical Analysis

The results are presented as the *mean* ± standard deviation (*SD*). Statistical analysis was performed using GraphPad Prism 9.5, and comparisons between groups were conducted using one-way analysis of variance (ANOVA) with Tukey’s post hoc test for multiple comparisons. Statistical significance was defined as *p* < 0.05.

## Results

### AAV Vector Carrying Ezh2 shRNA Was Expressed Whole DG of Hippocampus

To evaluate the effect of Ezh2 knockdown on neurogenesis after global ischemia, we used RNA-mediated gene silencing system delivered by AAV vector into DG. To evaluate the efficacy of Ezh2 shRNA, we first expressed four different Ezh2 shRNA constructs in gerbil fibroma cells. Transduction of Ezh2 shRNA-4 was the most effective among the four constructs and decreased endogenous Ezh2 expression by 60 to 70% (Fig. 1b). We used Ezh2 shRNA-4 for all experiments. By using this construct, we injected AAV vector carrying Ezh2 shRNA into the DG. We found that GFP signals were expressed both in the cell bodies and neurites of the whole DG 5 weeks after stereotaxic injection targeting DG, suggesting that shRNA system worked in the whole DG (Fig. 1d). Also, immunohistochemistry revealed that Ezh2 expression was consistent with pyramidal cell layer of CA1 and CA3 regions and GCL of DG (Supplementary Figure). Because the pyramidal neurons of CA1 region were lost after transient ischemia which is known as “delayed neuronal death,” [23] Ezh2 signals of the ischemia groups were also lost in the CA1 region.

### Ezh2 Knockdown Abolished Increment of Neurogenesis After Transient Forebrain Ischemia in DG

Next, we quantified the number of immunoreactive cells for EdU, which was administered 1 week after transient ischemia to label proliferating cells. EdU-positive cells were found both in the GCL and the subgranular zone (SGZ) of DG (Fig. 2a). The number of EdU-positive cells after sham operations did not alter both in the negative shRNA (14.8 ± 1.4) and Ezh2 shRNA (17.0 ± 2.2) groups, compared to the control group (20.4 ± 3.1; Fig. 2b). In contrast, transient forebrain ischemia significantly increased the number of EdU-positive cells of the negative shRNA group (64.2 ± 6.1, *p* < 0.001). Remarkably, we found that Ezh2 shRNA reduced the increment of EdU-positive cells after transient forebrain ischemia (33.0 ± 5.8, not significant vs control group, *p* < 0.05 vs negative shRNA + ischemia group).

Furthermore, we measured the distance from SGZ to EdU-positive cells to elucidate the effect of Ezh2 knockdown on the differentiation status of the neural stem cells. After the sham operations, the distance between SGZ and EdU-positive cells did not show significant difference in the negative shRNA (8.3 ± 0.9 μm) and Ezh2 shRNA (6.0 ± 1.0 μm) groups, compared to the control group (6.3 ± 1.5 μm). After transient forebrain ischemia, although not significant, the EdU-positive cells of the negative shRNA group showed longer distance from SGZ (11.5 ± 1.8 μm), compared to the control. On the other hand, Ezh2 shRNA + ischemia group (19.8 ± 2.5 μm) showed significantly longer distance from SGZ compared to the control (*p* < 0.001) and even negative shRNA + ischemia (*p* < 0.05) groups.

These data suggested that, specifically after ischemia, Ezh2 knockdown shifted the balance between self-renewal and differentiation toward differentiation in adult DG.

### Ezh2 Knockdown Decreased Neural Stem Cells in SGZ After Forebrain Ischemia

To evaluate the neural stem cells after forebrain ischemia in SGZ, we quantified the number of immunoreactive cells for Sox2 in SGZ, which is a marker for neural stem cells and immature neurons [24]. The number of Sox2-positive cells after sham operations did not alter both in the negative shRNA (309.4 ± 7.7) and Ezh2 shRNA (324.4 ± 16.5) groups, compared to the control group (333.8 ± 23.9; Fig. 3). Transient forebrain ischemia significantly decreased the number of Sox2-positive cells of the negative shRNA (242.0 ± 34.5, *p* < 0.05) and Ezh2 shRNA (210.4 ± 10.2, *p* < 0.01) both.

These data suggested that neural stem cells in SGZ decreased after global ischemia, and, specifically after ischemia, Ezh2 knockdown further decreased neural stem cells in SGZ.

## Discussion

Our study showed that AAV-mediated Ezh2 knockdown in DG significantly decreased the ischemia-induced increment of proliferating cells, and the proliferated cells after ischemia showed significantly longer migration from SGZ in the Ezh2 shRNA group compared to the control group although post-ischemic migration in the negative shRNA group also showed longer tendency which was not significant (Fig. 2). Of note, in the non-ischemic status, the number of proliferating cells and the distance of proliferating cells from SGZ did not show any difference between negative shRNA and Ezh2 knockdown groups. Furthermore, the number of neural stem cells of SGZ significantly decreased in the ischemia plus negative shRNA group compared to the control, and further decreased in the ischemia plus Ezh2 knockdown group (Fig. 3). The role of Ezh2 has been investigated especially in the stem cell proliferation and neurogenesis during embryo. However, there are few reports focused on the role of Ezh2 in the adulthood. To our knowledge, this study is the first report to show the regulatory role of Ezh2 in adult neurogenesis of DG in neurodegenerative status.

**Fig. 4:**
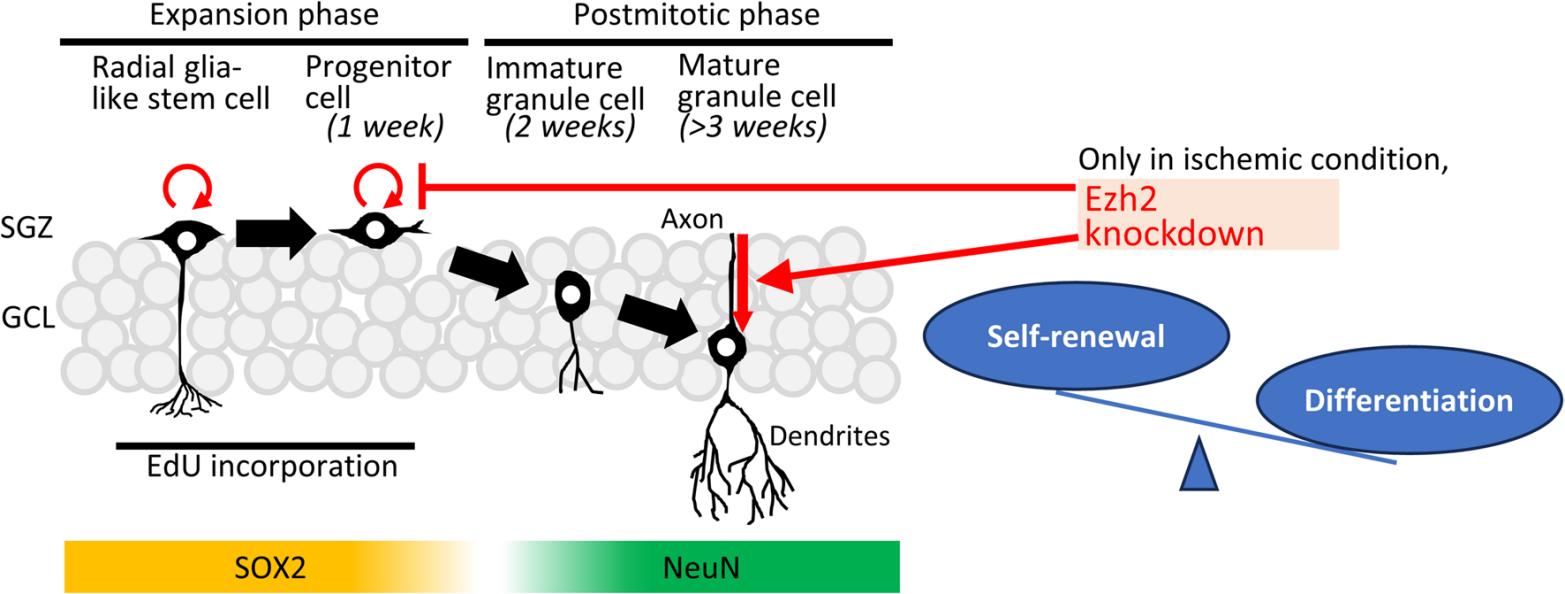
The concept of this study. After birth, neural progenitor cells in SGZ are capable of proliferation and multi-potential differentiation but are unable to self-renew. One week after birth, these progenitors are committed to neuronal lineage. Two weeks after birth, they differentiate and migrate into GCL. It takes about 2 months from birth of neural progenitor cells to mature granule cells fully incorporated into the hippocampal circuits. Our data suggested that, specifically after ischemia, Ezh2 knockdown shifted the balance between self-renewal and differentiation toward differentiation in adult DG. EdU, 5-ethynyl-2′-deoxyuridine; GCL, granule cell layer; SGZ, subgranular zone

Adult neurogenesis persists in two discrete regions of SVZ and DG, which is increased by some neurodegenerative status such as ischemic insult [25, 26]. After ischemic insult, it has been reported that the number of proliferating neural stem cells in SGZ of DG increases with its peak at 7 days and gradually decreases to the control level [27–30]. In this study, we systemically administered thymidine analogue, EdU, 1 week after forebrain ischemia to label proliferating neural stem cells. The half-life of EdU in vivo is proven to be about 30 min [31], which means that the EdU-positive cells in this study proliferated in SGZ at 7 days after ischemia. After birth, neural progenitor cells in SGZ are capable of proliferation and multi-potential differentiation but are unable to self-renew (Fig. 4). One week after birth, these progenitors are committed to neuronal lineage. Two weeks after birth, they differentiate and migrate into GCL. It takes about 2 months from birth of neural progenitor cells to mature granule cells fully incorporated into the hippocampal circuits [11, 32]. Therefore, EdU-labeled cells in this study could be in their later immature granule cell stage because the immunohistological evaluation was performed 2 weeks after EdU administration. The limitation of this study is that the long-term fate of EdU-labeled cells is not elucidated because we did not have animal groups which were examined more than 2 weeks after EdU administration.

The process of neurogenesis seems precisely regulated, although much remains unknown about the regulatory factors which affect post-ischemic neurogenesis of adult DG. Post-ischemic increment of neurogenesis in DG is reported both after global and focal ischemia [27, 28, 33, 34]. Because the focal ischemia reproduces the pathophysiology of cerebral infarction which is different from cardiac arrest focused on in this study, here we cite previous studies on the regulatory factors of post-ischemic neurogenesis after global ischemia. Total protein levels of CPG15 and Sema4C and activity of caspase-3 and matrix metalloproteinases were reported to increase in hippocampus of adult rodents after global ischemia [35–38]. Also, phosphorylation of Mek, Erk, 90Rsk, and Msk levels were increased in hippocampus of adult gerbils after forebrain ischemia [39]. Bcl-2 overexpression driven by neuron-specific enolase promoter was shown to increase newborn neurons of DG after global ischemia in adult transgenic mice [40]. Administration of recombinant peptide of last nine amino acids of GluN2B carboxyl tail increased newborn neurons of DG after global ischemia in adult rats [41]. FABP7 knockout decreased new born neurons of DG both under physiological and ischemic conditions in adult mice [42]. In contrast to focal ischemia, not so many reports were found on the regulatory system of adult neurogenesis after global ischemia.

Ezh2 catalyzes H3K27me3, which is a site specific methylation of histone tails and suppresses gene expression by altering the accessibility of transcription factors to DNA [15]. Ezh2 is ubiquitously expressed in a wide range of cell types including neural stem cells of SVZ and DG [43]. Our immunohistochemical analysis also showed the broad expression of Ezh2 in hippocampus (Supplementary Figure). In the mice embryo, cortex-specific deletion of Ezh2 using Cre expression system resulted in a prominent upregulation of gene expression, which also showed overproduction of neurons in cortical plates and depletion of neural stem cells in SVZ by E16, the peak of neurogenesis [18]. In the adult mice, tamoxifen-induced conditional knockout of Ezh2 reduced proliferation of neural stem cells and generation of neurons in DG, resulting in impairment of learning and memory [44]. These two studies showed opposite results in generation of neurons under the Ezh2-deleted condition, possibly because the role of Ezh2 is different in embryo and adults. In our study, deletion of Ezh2 using AAV expression did not affect the cell proliferation in the sham-operated controls but abolished the post-ischemic increment of cell proliferation. Furthermore, Ezh2 knockdown did not affect the distance from SGZ to proliferated cells in the sham-operated controls but significantly increased in the ischemia groups (Fig. 2). Our results indicated that, specifically after ischemia, Ezh2 knockdown shifted the balance between self-renewal and differentiation toward differentiation in adult DG. Recent study showed that although embryonic knockdown of Ezh2 in neural stem cells showed little to no cell fate transitions, the double knockout of both Ezh2 and Suv4-20h caused a dramatic defect of hippocampal development [45]. Suv4-20h catalyzes trimethylation of histone 4 lysine 20 and regulates hippocampal neurogenesis through posttranslational modifications [43]. Similar to our results, the study also shows that change of the downstream gene expression by Ezh2 single deletion may not be enough to affect neurogenesis. In the future studies, transcriptome analysis of DG after ischemia can be helpful to identify the precise regulatory mechanisms of Ezh2 on post-ischemic neurogenesis.

We used the AAV-based system to knockdown Ezh2 in this study. Due to its non-pathogenicity and efficacy, AAV vectors are now widely used for experimental and clinical purposes [46–48]. AAV is a non-enveloped, single-stranded DNA virus with its diameter of 20–25 nm and does not replicate in the absence of helper viruses such as adenovirus or herpes virus [49]. Of note, the carrying capacity of AAV vectors should be less than 4.7 kb [50, 51]. To date, over one hundred natural variants of AAV have been identified, and natural serotypes of AAV1, 2, 5, 8, 9, and rh10 are the most commonly used for central nervous system applications [52]. Most AAV serotypes are known to transduce genes predominantly into neurons; some serotypes also transduce astrocytes, oligodendrocytes, and endothelial cells [50]. Because the transduction efficiency of AAVs into neural stem cells had not been precisely investigated, we recently reported that AAV5 showed the highest transduction efficiency into neural stem cells among AAV2, 5, and rh10 [53]. Because AAVrh10 showed the second highest efficiency into neural stem cells and the highest into neurons of GCL among these three serotypes in that report, we used AAVrh10 in this study. Different from other serotypes, AAV5 shows strong tropism to glia as shown in our previous articles [53, 54].

We found the post-ischemic decrement of neural stem cells in SGZ both in negative shRNA and Ezh2 shRNA groups (Fig. 3). We could not find previous studies which focused on the number of neural stem cells of SGZ after ischemia. Many studies so far have quantified the newly-generated neurons in GCL which were mostly labeled with thymidine analogue, such as bromodeoxyuridine [39, 55–58]. Because the post-ischemic decrement of neural stem cells in the negative shRNA group was significant compared to the control group but not so much, further research should be necessary for the establishment of neural stem cell loss in GCL after ischemia. On the other hand, the post-ischemic decrement in the Ezh2 shRNA group was prominent. As shown in the above-referred articles, Ezh2 deletion was reported to reduce the neural stem cells of SVZ in embryo [18] and the size of neurospheres isolated from adult DG [44], in which the common mechanisms may exist with our study.

## Supplementary Information

ESM 1 Supplemental Figure. Ezh2 was broadly expressed in DG. Because the pyramidal neurons of CA1 region were lost after transient forebrain ischemia, Ezh2 signals in the ischemia groups were also lost (white arrowheads). DAPI: 4',6-diamidino-2-phenylindole. Scale bar = 500 μm. (TIF 10053 kb)

## Data Availability

The original datasets presented in the current study are available from the corresponding author.
