# Supplementary figures and images for: Adeno-associated Virus-mediated Ezh2 Knockdown Reduced the Increment of Newborn Neurons Induced by Forebrain Ischemia in Gerbil Dentate Gyrus
Source: Mol Neurobiol. 2024 Apr 27;61(11):9623–32. doi: 10.1007/s12035-024-04200-w (PMC11496322; doi:10.1007/s12035-024-04200-w)

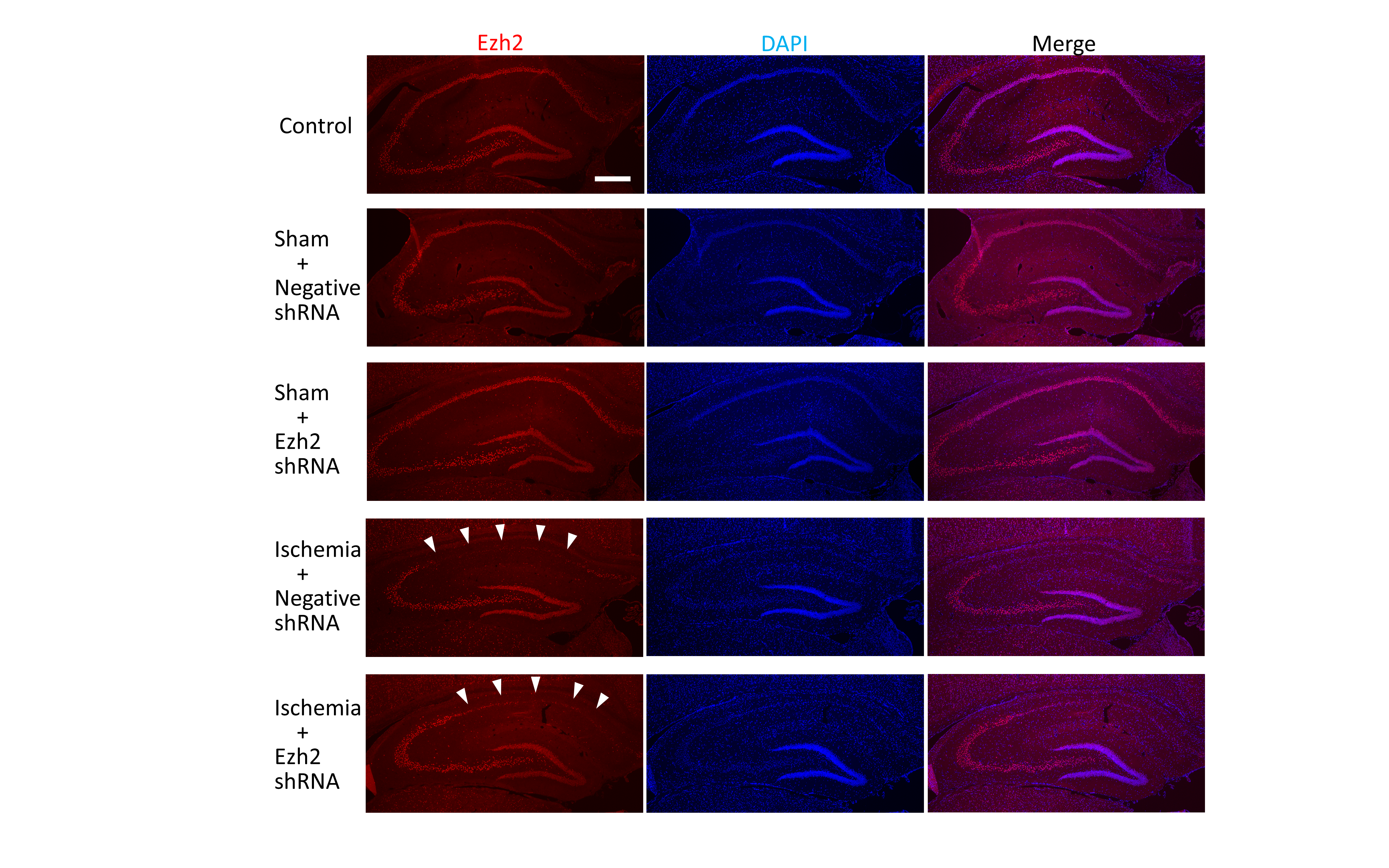

Supplement: Supplementary file 1 — Supplemental Figure. Ezh2 was broadly expressed in DG. Because the pyramidal neurons of CA1 region were lost after transient forebrain ischemia, Ezh2 signals in the ischemia groups were also lost (white arrowheads). DAPI: 4',6-diamidino-2-phenylindole. Scale bar = 500 μm. (TIF 10053 kb) [file 12035_2024_4200_MOESM1_ESM.tif]
